# On the Division of Medical Practice

**Published:** 1811-05

**Authors:** 


### *To the Editors of the Medical and Physical Journal.*

### *On the Division of Medical Practice.*

Gentlemen,

The observations of your Correspondent Censor, on the Contraction, or what he somewhat quaintly terms "the growing *stricture*" of the practice of the physician in London, appear to me, though I am not prepared to follow him in all his conclusions, to be peculiarly deserving of consideration at the present time, time, when such unceasing efforts are made to disparage the talents, and to vilify the characters of the most eminent medical practitioners. With the view of counteracting the impression which has been made in certain quarters, I had already committed to paper some remarks on the same subject; and as I am anxious to see the question fully and fairly discussed, I shall feel obliged if you will afford me the opportunity of communicating them through the medium of your Journal.

Nothing can be more absurd than the outcry which has been raised against the higher orders of the profession by a set of weak and inconsiderate men, who, coveting the fee of the physician, would bring back the ancient order of things, when medical practice was yet undivided—not being able to foresee, that such a change would have the inevitable effect of annulling this mode of payment altogether. Μιμειται που και φαρμακοπωλης ιατρον is an old complaint; but till 'Squire Jeremiah Jenkins* arose to illumine the world, I know not that any one has had the boldness to recommend, that "*the ridiculous title of Physician should be abolished,*" and supreme authority conferred upon the practising apothecary. Unfortunately, however, in his excess of zeal for the cause which he espouses, this champion of the pestle and mortar has been betrayed into certain inconsistencies, which completely frustrate all the vehemence of the attack. After, for example, asserting, that "the apothecary of this country *is qualified by education to attend at the bed-side of the sick,* and being in general better acquainted with pharmacy than the physicians of the English Universities, and not less versed in anatomy, physiology, and pathology, is often the most successful practitioner," he somehow or other admits, that "*not one half the apothecaries of London have a proper education,* and although not able to discriminate one disease from another, yet his [*i. e.* one half of the London apothecaries'] whole time is devoted to visiting patients, while the composition of the medicine is left to a careless young man, who is as ignorant of drugs as his master is of diseases, and who in the absence of his master will employ the errand-boy to compound the medicines.—More lives," he continues, "are sacrificed by the carelessness

* See a pamphlet entitled "*Observations on the present State of the Profession and Trade of Medicine, as practised by Physicians, Surgeons, Apothecaries, Chemists, Druggists, and Quacks, in the Metropolis, and throughout the country of Great Britain. By* JEREMIAH JENKINS, ESQ. *late Practitioner in Medicine.*" This name, there is every reason to believe, is fictitious; and the pamphlet itself is merely a reprint of papers inserted in a periodical Journal called "The Medical Spectator."

of of assistants, and *ignorance of apothecaries*, than are even prolonged by medicine, much more restored to health." But he very soon forgets this confession; for, a few pages further on, we find him observing, that "as experience makes the physician, in this respect many apothecaries *have a decided advantage over the graduates of Oxford and Cambridge*," and that "every gentleman that enters the profession as an apothecary, should look forward to the highest honours in medicine." Notwithstanding these extraordinary merits, Jeremiah Jenkins assures his readers, that "in the present state of medicine, the apothecaries of London are really not so well paid as *the common porters;* and it is hard indeed, if, after ten or twenty years laborious practice, he (*i. e.* the apothecaries of London) is not allowed to participate in the honours: but, as I happen not to be acquainted with any members of the worshipful company of porters, who ride about in their chariots, and indulge in all the luxuries of life, I am disposed to question the accuracy of this statement. I am not even without my suspicions that the whole performance is designed as a satire upon apothecaries; and in this idea I have been almost confirmed by a passage, in which the author suggests a very singular and novel expedient for alleviating the hardships he so pathetically laments. "Indeed," observes 'Squire Jenkins, "if the physician was a liberal man, he would DIVIDE THE FEE WITH THE APOTHECARY; and were the writer an apothecary in London, he would not call in a physician, if he really did not stand in need of his assistance, *unless he would agree to allow a moiety of the fee*."

To the surgical tribe this exquisite writer is much more complaisant. "The surgeon of the present day," he remarks, "is a friend to real science, and, generally speaking, not a slavish follower of the opinions of his teacher, but in all cases dares to think for himself. The surgeons of the London hospitals and the provincial infirmaries, are men of sound judgment, accurate reasoning, and acute observation: and *when the practice of physic is united to surgery, we may expect that medicine will make some progress to improvement*." And, in another place, he states his conviction, that, "if the surgeons who do not practise pharmacy were to prescribe draughts, pills, and bolusses, they would be more frequently called in by the apothecary than they are; and *his* opinion is, *that were they more consulted, we should hear less of organic diseases and their consequences*."

On these vague and unsubstantiated assertions I should think it superfluous to offer any comment, were it not that they lead to some considerations on which I am anxious to enlarge.

enlarge. Without entering into a discussion of the comparative qualifications of the surgeon and physician, I may be permitted to state my belief, that the advantages which result from the separation of the two branches of the art are great and important; and this opinion receives confirmation, if I mistake not, from every part of their history. As long as the physician continued to officiate in a double capacity, the improvement of surgical science was extremely slow: the moment that surgery became a distinct pursuit, and an honourable employment, it advanced with great rapidity. To what, for instance, are we to ascribe the superior eminence and skill for which the French surgeons were so deservedly celebrated, but to the early establishment of the College of St. Côme, which placed them on a level with the Medical Faculty, and gave to their art a dignity and an importance to which it had been till then a stranger? To what are we to refer the accelerated progress of surgery in this country, during the last century, but to its rescue from the disgraceful connection in which it had been held with a low mechanical trade? In those countries, on the other hand, where this emancipation has taken place only in an imperfect degree, as in Holland and Germany, the surgical art has not, till within these very few years, been cultivated with any degree of success; and that now, perhaps, more from the incitement of surrounding examples, than from a native spirit of improvement.

"The surgeons of the London hospitals and the provincial infirmaries," however, "are men of sound judgment, accurate reasoning, and acute observation." Undoubtedly they are so; and they will admit, that I am pleading their cause when I maintain, that it is chiefly owing to this fortunate subdivision of practice that they have arrived at their present high professional skill. Would a Bromfield, a Pott, or a Hunter, ever have so far outshone their cotemporaries, if they had not opened, as it were, for themselves, a new and isolated career. What branch of surgery, or what province of medicine, would have been improved by their labours, if they had pursued a different course; and, if, in the eagerness of emolument, they had endeavoured to grasp the whole compass of medical learning?—But, "if *surgeons* were more consulted, we should hear less of *organic* diseases." Contemptible insinuation, the effusion of ignorance and malice!—as if *physicians* were forward in inventing disorders which have no real existence; and as if every day's experience did not furnish fresh proofs that the pathology of the viscera is unhappily but too little understood. The authors of of such slanders, however, cultivate no pathology, no morbid anatomy; physiological science is far removed from them: their chief study is detraction; and their distinguishing qualities are impudence and presumptuousness.

In attempting to fix the line of demarkation between physic and surgery, I shall probably expose myself to the imputation of being actuated by interested motives: but I feel that I am only supporting the common rights of my brethren, and know, that I shall be seconded by every liberal and enlightened surgeon in making a stand against the factious and turbulent designs of those medicasters, who, in the sordid spirit of gain, would sacrifice all that is great and dignified in the art. I have heard surgeons themselves complain, that their province was not sufficiently circumscribed, or that its boundaries were too often transgressed; I have even heard some of them acknowledge, that they had no prescriptive right to the treatment of certain classes of diseases which custom has now generally assigned to them: and yet, if the physician is caught tripping, a thousand tongues are ready to decry his rapaciousness. Shall the surgeon, then, be permitted quietly to invade the possessions, and to seize upon whatever share he may covet, of the rightful inheritance of the physician? Far be it from me to deprive him of any description of practice, which he may have made his particular study, and in which he may have acquired merited fame; but I repeat, that it is for the mutual interest of the two departments that their limits, being once determined, should be strictly adhered to. If the surgeons will evade the law, let the physicians have equal justice; and if the former will insist on prescribing for hysterics and nervous fever, let the latter resume their authority over syphilis and scrofula. A very slight degree of reflection is sufficient to shew, that medicine presents such an extensive field for exertion, that to cultivate it with equal success in all its various parts is beyond the reach of any individual, however great his genius, however vast his powers. Accordingly, as soon as any one is observed to have paid particular attention to this or that class of complaints, or to have studied only with more than common care the structure and morbid alterations of a single organ of the body, he is sure to obtain a run of employment in that department, to the exclusion of all other practitioners whatever. At the time that Mr. Hunter was at the height of his fame, would any one have submitted their eyes to be couched by him in preference to Baron Wenzel: and at the present day, what man in his senses would confide the repairs of his teeth or his nails to the most experienced general operator, while a dentist or corn-cutter were at hand?

In short, that division of medical practice which has now generally obtained throughout the greater part of Europe, has always appeared to me to be sanctioned by obvious expediency, and to conduce to the advantage not only of the art itself, but of all who become the objects of its care. Like the division of labour, which, in the mechanical arts, facilitates and expedites so highly the operations of industry, it enables the physician, the surgeon, and the pharmacist, to become each more expert in his respective province, and to furnish society with more skilful workmanship, than could be performed, if the three branches of the trade were, as formerly, united. The increasing labour and expensiveness of medical study, the general progress of civilization required such a division; and they are the enemies of every thing that is scientific and respectable in the profession, who would wish to see it done away.

I am, Gentlemen,

A PHYSICIAN.

*London, April* 12.

